# Commercial vision sensors and AI-based pose estimation frameworks for markerless motion analysis in sports and exercises: a mini review

**DOI:** 10.3389/fphys.2025.1649330

**Published:** 2025-08-12

**Authors:** Saeid Edriss, Cristian Romagnoli, Lucio Caprioli, Vincenzo Bonaiuto, Elvira Padua, Giuseppe Annino

**Affiliations:** ^1^ Department of Industrial Engineering, Sports Engineering Laboratory, University of Rome Tor Vergata, Rome, Italy; ^2^ Department of Human Science and Promotion of Quality of Life, Human Performance, Sport Training, Health Education Laboratory, San Raffaele Open University, Rome, Italy; ^3^ Department of Medicine Systems, Human Performance Laboratory, Centre of Space Bio-Medicine, University of Rome Tor Vergata, Rome, Italy

**Keywords:** markerless motion capture, vision sensors, pose estimation, human movement analysis, sports biomechanics, kinematic analysis, artificial intelligence in sports, sports technology

## Abstract

Kinematic and biomechanical analysis in monitoring human movement to assess athletes’ or patients’ motor control behaviors. Traditional motion capture systems provide high accuracy but are expensive and complex for the public. Recent advancements in markerless systems using videos captured with commercial RGB, depth, and infrared cameras, such as Microsoft Kinect, StereoLabs ZED Camera, and Intel RealSense, enable the acquisition of high-quality videos for 2D and 3D kinematic analyses. On the other hand, open-source frameworks like OpenPose, MediaPipe, AlphaPose, and DensePose are the new generation of 2D or 3D mesh-based markerless motion tools that utilize standard cameras in motion analysis through real-time and offline pose estimation models in sports, clinical, and gaming applications. The review examined studies that focused on the validity and reliability of these technologies compared to gold-standard systems, specifically in sports and exercise applications. Additionally, it discusses the optimal setup and perspectives for achieving accurate results in these studies. The findings suggest that 2D systems offer economic and straightforward solutions, but they still face limitations in capturing out-of-plane movements and environmental factors. Merging vision sensors with built-in artificial intelligence and machine learning software to create 2D-to-3D pose estimation is highlighted as a promising method to address these challenges, supporting the broader adoption of markerless motion analysis in future kinematic and biomechanical research.

## 1 Introduction

Understanding musculoskeletal movement is an important approach in monitoring human movements and motor behaviors (Schmidt and Lee, 2019). Kinematic and biomechanical analysis helps evaluate these parameters to measure and capture the degrees of freedom of joints and limbs independently, diagnose injuries, and analyze the performance of athletes and patients (Dao and Tho, 2014). Numerous studies using motion capture systems (MoCap), wearable sensors, and technologies have become essential tools in laboratories and field tests for investigating specific biomechanical or kinematic issues. While these technologies serve as the gold standard for monitoring individuals in 3D simulations, their high costs and the setup complexity present significant limitations (Edriss et al., 2024a; Patrizi et al., 2016).

In the recent decade, body simulation in 2D and 3D has undergone rapid evolution, driven by artificial intelligence (AI), and has combined with the sports, medicine, animation, and gaming industries (Khan et al., 2024; Dai and Li, 2024). Kinematic monitoring and analysis represent a contemporary method for simulating human body motion, especially in 2D (Seo et al., 2019). Numerous studies have examined the validity of various devices and software designed to monitor specific motions. While 3D data offers more information than 2D data, the choice largely depends on the intended application. 2D information can be suitable for particular applications, especially concerning ease of use and cost. However, 2D simulation has limitations like restricted access to certain angles and the capturing of movements from specific perspectives. Despite these limitations, these systems provide significant advantages, including low costs for registering videos with one or a few cameras, quick and easy to setup and operate (Caprioli et al., 2024). Kinematics is valuable for characterizing changes in specific joint angles to monitor how motion performs and loads in land and water environments (Cronin et al., 2019). Markerless systems use RGB, depth, or infrared cameras, with Microsoft launching the successful commercial system supported by a pose estimation (PE) device in 2010 (Holte et al., 2012). Now, vision sensors, including depth RGB-D and stereo cameras, are being used. Depth RGB cameras capture actual 3D spatial information through active sensing methods such as infrared projection, stereo vision, or Time-of-Flight technology, and stereo cameras use two RGB lenses to estimate depth through disparity. Later, in 2017, OpenPose, as one of the first PE frameworks, was released to extract body pose from standard 2D videos (Qiao et al., 2017).

This review explores the development of commercial technologies in real-time and offline markerless MoCap, starting with earlier systems using vision sensors that enable direct 3D motion tracking, followed by newer PE frameworks that analyze standard videos with AI, emphasizing the importance of their integration. Each methodology has its exclusive capabilities, and familiarity with each tool is beneficial; however, recent research has increasingly combined both to enhance functionality and usability. Conversely, PE frameworks estimate joint positions from 2D RGB videos using machine learning (ML) algorithms. Consequently, studies since 2010 on kinematic hardware and software for 2D and 3D analysis have focused on their validity and reliability compared to gold-standard systems. The selected experimental validation and technical reports focused on body motion in English are sourced from IEEE, PubMed, and Google Scholar since 2012, with PE tools from 2018 onward. The following keywords and combinations were used: “markerless-MoCap”, “vision-sensors”, “pose-estimation”, “MediaPipe”, “OpenPose”, “RealSense”, “Kinect”, “ZED-camera”, and “sports-tracking-analysis.” The review also highlights the chosen gold-standards for these systems’ validation, optimal perspectives, and setups to achieve the best results in sports applications, emphasizing the practical benefits and uses of these technologies in human activity research and athletic performance, and emphasizing the need for future vision sensor generation to integrate AI-based PE frameworks for broader usability.

## 2 Hardware (vision sensors)

### 2.1 Kinect

Microsoft designed and released Kinect K1 (in 2010) and K2 (in 2014), which consist of one IR emitter, one IR camera, and one RGB camera, to gain depth and color images. To strongly enter the gaming industry and provide human connections as a part of Xbox consoles, targeted the companies that designed the Kinect, which has lately been used as a markerless, affordable, and portable MoCap sensor (Bilesan et al., 2019). Kinect can perform PE using its built-in depth sensor and the associated software development kits (SDKs). Many studies explored the accuracy of the two Kinects in real-time skeletal tracking during human movements (Ganea et al., 2014; Kurillo et al., 2022). However, according to (Wang et al., 2015), regarding MoCap selection as a gold standard, the K2 generation is beneficial in terms of joint estimation accuracy and more robust in handling track occlusion and body rotation. In another study, a Vicon MoCap was compared with Kinect in gait analysis, as participants walked and jogged on a treadmill. The results show the validity of the Kinect in the sagittal plane for stride timing in kinematic analysis (Pfister et al., 2014).

Depending on the coaches’ purpose, estimated pose through the Kinect somatosensory camera can be sufficient in physical activity recognition, such as qigong movement detection (Fan et al., 2024). Or by requiring more complex analysis processes, their implementation with other codes or devices, such as 3DSMAX, can invent novel methods for monitoring the sports training seasons (Shi, 2014). Additionally, regarding simulating and estimating the body joints and limbs, the skeletal body structure, Asteriadis et al. used Kinect in body estimation in 3D by minimizing the noise (Asteriadis et al., 2013).

The Kinect device alone lacks accuracy in defining orthogonal axes unrelated to joint centers, limiting its ability to measure internal or external angular rotations and displacement. Adding depth sensors, video cameras, or multiple devices to make a MoCap-based Kinect can improve the tracking process and angular measurement accuracy (Clark et al., 2012; Napoli et al., 2017).

### 2.2 StereoLabs ZED camera

The ZED Camera by StereoLabs, released in 2015, was followed by the next-generation, including ZED2 (2019) and ZED2i (2021), which were enhanced with depth sensing and AI features (Sarıalioğlu et al., 2024). ZED cameras are equipped with two high-resolution lenses to capture in-depth information, making them suitable commercial vision hardware for integrating open pose libraries in kinematic and biomechanical studies (Avogaro et al., 2023). A comparative analysis of the ZED2 camera with other commercial vision systems, including Azure Kinect and RealSense D455, utilizing their respective SDKs, shows higher performance in human pose tracking at distances exceeding 3 m. This advantage aids PE in larger spaces or dynamic environments, such as field tests (Ramasubramanian et al., 2024). Another suggests that Nuitrack and MediaPipe software integrate with ZED2i to perform better in upper and lower extremity features for pose tracking (Aharony et al., 2024). Combining ZEDmini with a Virtual Reality (VR) headset and an augmented reality (AR) prototype resulted in a device capable of recognizing body formation and sports equipment such as a tennis racket and basketball hoop. This novelty aids players with low vision to enhance their ability to identify the players and objects (Lee et al., 2024). The advantages of ZED cameras as hardware for exercise monitoring, assessing gait impairment in Neurodegenerative disorders, and joint angle measurement include their ease of use, affordability, and ability to deliver reliable results in both 2D and 3D kinematic analyses of exercises, such as squats (Aliprandi et al., 2023; Zanela et al., 2022).

### 2.3 Intel RealSense

Intel RealSense cameras combine high-resolution RGB imaging with depth perception, offering models like the D400 series for general depth sensing, the L515 for precise LiDAR tasks, and the T265 for motion tracking with simultaneous localization and mapping (SLAM) capabilities (Moghari et al., 2024; Tsykunov et al., 2020). By capturing depth maps and RGB frames, they integrate seamlessly with pose-estimation software framework tools, mapping 2D keypoints to real-world coordinates. Their lightweight design, robust SDK, and real-time performance make them versatile for indoor and outdoor applications, outperforming infrared-based systems like Kinect in varied lighting conditions (Maddipatla et al., 2023).

An Intel RealSense camera is used in head motion tracking using custom software while participants walk indoors and outdoors. They walked in both slow and fast modes, and their head motion tracking was compared with that of a perambulator containing inertial measurement units (IMUs) and a distance counter, serving as the gold standard. The accurate results of this gait study showed that while the participants walked slowly (Hausamann et al., 2021). Another study compared the Intel RealSense camera to the Kinect in slow walking gait analysis. The results indicated that while the Intel RealSense D435 is valid for measuring certain spatiotemporal variables, the Kinect proved more reliable for collecting skeletal data due to its more robust RGB-D camera (Mejia-Trujillo et al., 2019). MediaPipe landmarks and two Intel RealSense cameras were used to estimate angular parameters in five exercises, including squats, knee, ankle, hip extension, and shoulder elevation, compared with OptiTrack as the reference. The results showed an error of less than 20 degrees, demonstrating an adequate estimation quality. Still, further improvements in both software and hardware can enhance the system’s accuracy (Pilla-Barroso et al., 2024). These generations of cameras are increasingly being used in sports performance analysis through integration with AI-based markerless MoCap software. Intel RealSense depth cameras serve as sufficient registration tools in markerless PE, especially for closer range (under 3 m) (Ramasubramanian et al., 2024; Jacobsson et al., 2023).

## 3 Software (pose estimation models)

### 3.1 OpenPose

OpenPose is an open-source library developed by Carnegie Mellon University for 2D PE of the skeletal structure by detecting 25 keypoints on the body (Hidalgo et al., 2019). A real-time detection system may focus on specific angles between joints, the center of mass, or a point of displacement (Cronin et al., 2024; Needham et al., 2021). To analyze the running performance and timing, OpenPose evaluated it with the Coco dataset regarding human PE to recognize hurdles athletes’ wrists, ankles, hips, and knees (Jafarzadeh et al., 2021; Sharma et al., 2022). The Coco dataset recognizes 18 pre-trained landmarks with x, y, and v values, where x and y indicate coordinates, and v shows the visibility of the landmarks (Duan et al., 2023).

PE relies on video quality and frame rates, meaning that weather conditions and lighting impact the accuracy of ML systems. A study evaluating the AI accuracy of OpenPose as a PE model under various environmental and lighting conditions, while participants performed stretching exercises, found that the accuracy rates depend on the environment and setup context (Song and Chen, 2024).

In a study using 3D motion analysis as the reference standard, an OpenPose-based motion analysis was used to compare knee valgus angular data during the drop vertical jump test. The strong correlation among these data indicated a sufficient ML accuracy level of the PE models in measuring angular movements (Ino et al., 2024). In baseball, analyzing a hitter’s limb angles and hip distance aids in swing performance (Li et al., 2021). AI-driven gesture recognition supports biomechanical analysis in training techniques and game strategy evaluation. This approach was a key objective in a study analyzing tennis player performance through skeletal PE (Wu et al., 2023).

Furthermore, PE models’ integration with wearable sensors or MoCap can facilitate more complex processes through Taekwondo kinematic analysis through a PE model, 8 body joint angles in players’ upper and lower body registered by Contemplas cameras, and the Vicon system. OpenPose was used to process recordings from Contemplas cameras to extract 2D human skeleton data, then triangulated to obtain 3D joint angles to compare world-class and master-class players’ kicking performance (Fukushima et al., 2024). Plus, optimal and kinetic analysis of fouling and shooting in basketball player performance was conducted using video-based (2D) methods with a lightweight deep learning (DL) architecture, where OpenPose was used as the PE model (Xu, 2024). This open-source framework is being used in athletes’ 3D estimating with integration with sensors or the Internet of Things (IoT) in various sports such as gymnastics (Ren et al., 2025).

### 3.2 MediaPipe

MediaPipe is an open-source framework developed by Google for 2D human PE (Pham et al., 2022). It enhances AI applications by detecting keypoints on the body, face, and hands in images or live video streams to offer gesture recognition, motion analysis, and interactive systems (Saini, 2024). MediaPipe is a broader framework that includes BlazePose and other ML solutions, where BlazePose specializes in detecting 33 keypoints (Wang, 2024).

An important finding shows that when assessing PE quality using MediaPipe from a frontal and lateral view, an error margin of less than 25% of the range of motion is optimal, with specific tolerances: under 20 degrees for movements greater than 90°, around 10 degrees for movements up to 40°, and below 10° for static angles (Pilla-Barroso et al., 2024).

The validity of MediaPipe BlazePose in measuring joint angles compared with the IMU-based MoCap in 2D image analysis, and the results showed that the difference in accuracy is within 10% (Pau et al., 2021). In addition, an ML tool using MediaPipe simulated kinematic points, assuming shoulder, knee, hip, and ankle joints for artistic swimming analysis. Regarding the 2D kinematic simulation frames, the leg deviation angles were measured during execution and body position in preparation to aid officiating systems and performance monitoring (Edriss et al., 2024b).

In a video performance analysis using an ML tool with MediaPipe, the knee angles of advanced and beginner pickleball players were measured during dink shots. This kinematic analysis assessed the differences in knee bending and body flexion between the two groups, suggesting an appropriate positioning formation and preparation for dinking (Edriss et al., 2025).

An application using MediaPipe, compared with Kinect, was used to measure knee angles for athletes under a motion test. The results showed the higher capacity of the MediaPipe framework in analyzing ACL injury risk by calculating lower limb kinematics through a smartphone (Babouras et al., 2024). However, a limitation of the MediaPipe source is the lack of ability to recognize multiple people in a frame (Dong and Yan, 2024).

### 3.3 AlphaPose

AlphaPose is an open-source tool that provides PE by detecting 25 keypoints. It was developed by the Chinese University of Hong Kong (Fang et al., 2023). Like OpenPose, AlphaPose provides a high keypoint detection rate and is highly interchangeable with ML models for biomechanical PE parameters like ground reaction forces (Mundt et al., 2023).

This framework is used in various ways, including gait and player kinematic analyses. For example, in gait analysis, participants walked at different speeds, and their estimated poses were captured using AlphaPose (Wei et al., 2023). Additionally, AlphaPose captured accurate skeleton PE of badminton players to classify their movements. These results achieved an 80% prediction accuracy, effectively analyzing the sequential nature of badminton actions (Liang and Nyamasvisva, 2023).

Several researchers are working on PE framework development through the DL tool, such as YOLO (Yang et al., 2024; Zhao et al., 2024). Although the validity of AlphaPose is sufficient, its accuracy compared to the data of other frameworks is considered. For example, a study compared MediaPipe BlazePose and AlphaPose with Vicon MoCap as the reference standard in gait joint kinematics analysis. This study emphasized that MediaPipe shows a lower mean square error (RMSE), indicating more accurate joint kinematic measurements. However, AlphaPose showed greater variability with a higher range of motion and RMSE (Hulleck et al., 2023).

### 3.4 DensePose

DensePose is a PE model developed by Facebook AI Research that maps human pixels in an image to the body’s 3D surface (Gu et al., 2022; Lovanshi and Tiwari, 2022; Mahajan et al., 2023). The advantages of DensePose, compared to other frameworks, are its perfect ability for multi-person recognition and its capability to estimate the body by keypoints or mesh (Güler et al., 2018; Guler and Kokkinos, 2019; Zhang et al., 2021). DensePose is effective for applications in augmented reality, virtual try-on, and immersive human-body interaction scenarios (Islam et al., 2024; Zhu and Song, 2021). Athletes’ biomechanical and kinematic analyses can be conducted by designing a 2D PE from videos using DensePose, which is pre-trained on the COCO keypoint dataset. For monocular 3D PE, MotionAGFormer, pre-trained on the Human3.6M dataset, can be utilized. DensePose requires more technical skills to set up due to its powerful GPU resources and custom code for mesh mapping. Studies indicate that joint angular measurements obtained through DensePose also provide reliable data (Hellstén et al., 2022; Suzuki et al., 2024).

**FIGURE 1:**
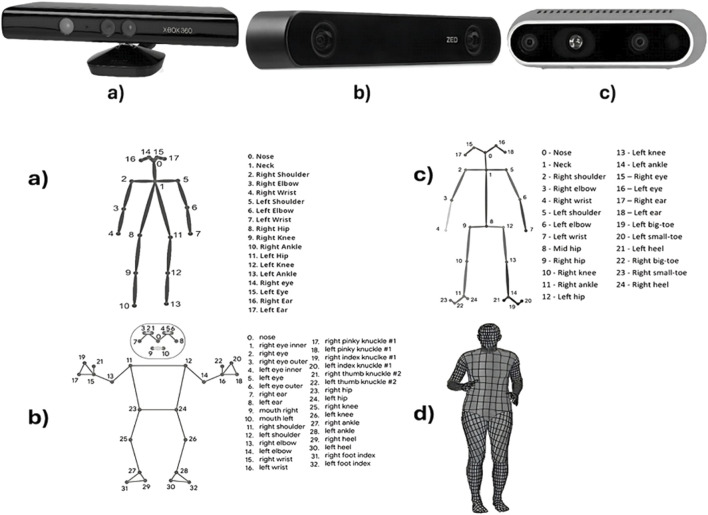
Top: Three types of depth cameras labeled **(a)** a Kinect for an Xbox 360 gaming console, **(b)** a ZED Camera by StereoLabs, and **(c)** an Intel RealSense depth camera model. Bottom: Three human body models corresponding to each camera, labeled **(a)** AlphaPose, **(b)** MediaPipe, **(c)** OpenPose, with numbered keypoints like nose, neck, shoulders, and more. Model **(d)** DensePose shows a mesh grid full-body representation.

Figure 1i illustrates the shape of the vision sensors, and Figure 1ii demonstrates the human skeletal structure as detected by the PE models.

Table 1 shows a brief comparison between hardware and software to achieve a clearer idea of their strength and pros.

**TABLE 1:** Provides a comparative overview of depth cameras and pose estimation frameworks, reviewing their typical error margins, envir onmental constraints, reliability, and suitability for recording players’ performance, with a focus on body limbs, setup complexity, and suitability for recording.

System	Type	Error range	Environment suitability	Depth range (m)	2D/3d output	Reliability (limb Focus)	Setup complexity	Key strengths and limitations
Kinect v2	Depth Camera	∼10°–20° (Pfister et al., 2014)	Indoor only	0.5–4.5 (Kurillo et al., 2022)	3D	Both (higher in the sagittal plane)	Low	Built-in skeletal tracking
ZED 2i	Stereo Camera	∼5°–15° (Aharony et al., 2024)	Indoor/Outdoor, >3 m	0.2–20 (Aharony et al., 2024)	3D	Lower limb (wide FOV applications)	Medium-High	Higher performance for wide views
RealSense	Depth Camera	∼10°–20° (Pilla-Barroso et al., 2024)	Indoor/Outdoor	0.2–10 (Aharony et al., 2024)	2D/3D	Lower limb (slow gait, squat)	Medium	Valid for close-range tracking
OpenPose	Software	∼5°–15° (Ino et al., 2024)	Indoor/Outdoor	Camera-limited	2D (pseudo-3D)	Upper limb (fine movement detection)	Medium	Good multi-person support
MediaPipe	Software	∼<10° (Pilla-Barroso et al., 2024)	Indoor/Outdoor	Camera-limited	2D	Lower limb (squats, gait)	Low	Fastest runtime, low memory use, suitable for mobile apps
AlphaPose	Software	∼15°–25° (Hulleck et al., 2023)	Indoor/Outdoor	Camera-limited	2D	Upper limb (combat/sport gestures)	Medium	Higher RMSE in some studies
DensePose	Software	∼10°–20° (Suzuki et al., 2024)	Indoor preferred	Camera-limited	2D/3D mesh	Both (AR/VR mesh mapping)	High	Ideal for multi-person and VR/AR

The numbers refer to the references mentioned in the text.

## 4 Discussion

Many researchers believe future sports motion studies will rely on markerless motion analysis systems (Ismail et al., 2016). Therefore, much research highlighted the accuracy and reliability of the body pose simulation device and software in the kinematic analysis procedures (Clark et al., 2012), particularly for motions within a single plane. Mainly, the articles validated body simulation tools by gold-standard IMUs or MoCaps such as Vicon or OptiTrack, comparing quantitative methods like RMSE in joint angles and joint position errors. Some studies applied qualitative validation, such as expert visual assessment or usability scoring. However, high limitations exist because 2D PE restricts their ability to capture out-of-plane movements, such as joint flexion or rotation, as they lack depth information (Pfister et al., 2014; Annino et al., 2023). Additionally, proper setup requires careful camera or device setup procedures to reduce their limitation (Yang and Park, 2024). Plus, PE accuracy depends on video quality, frame rates, and environmental conditions (outdoor, indoor, or natural or artificial lights), with studies showing varying OpenPose performance across different scenarios (Song and Chen, 2024).

Despite these drawbacks, 2D PE methods are beneficial due to affordability, ease of use, and relatively simple setup compared to 3D systems such as Vicon (Menychtas et al., 2023). Regarding the investigation aims, the camera and device can be set preferably if the motions are in the same plane, and it is beneficial to use the 2D PE methods to assess clinical or physical kinematic or biomechanical aspects (Gozlan et al., 2024; Hamill et al., 2012; Winter, 2009).

Regarding the articles, each hardware and software tool has limitations. Kinect and RealSense are constrained by lighting conditions, occlusions, and limited field of view, especially in dynamic environments. ZED cameras may require more computational resources. OpenPose and MediaPipe rely heavily on camera angles and lighting and may struggle with occlusions or out-of-plane movements. AlphaPose exhibits higher RMSE and variability in certain tasks, while DensePose requires significant computational resources. While these hardware and software tools can function independently, leveraging RGB video and PE software (for example, Kinect with OpenPose or ZED with MediaPipe) offers a simple way to obtain 3D body landmarks and points of interest for the upper and lower body analysis, with high accuracy (Ramasubramanian et al., 2024; Liu and Chang, 2022).

## 5 Conclusion

PE software has strengths compared to others; thus, selecting the best framework is not straightforward. For instance, some research illustrated MediaPipe’s advantage over OpenPose in offering lower deviation in keypoints detection, especially in feet and wrists, along with faster runtime (Latyshev et al., 2024). On the other hand, another study emphasizes OpenPose’s superiority over MediaPipe BlazePose in accurately detecting clinically relevant keypoints closer to anatomical joint centers (Mroz et al., 2021). In conclusion, while both models show promise, further improvements are necessary, and future studies will increasingly rely on AI-based markerless PE tools.

These methods provide a cost-effective and practical solution for studies focusing on movements confined to a single plane. They are particularly beneficial for assessing clinical, physical, and kinematic or biomechanical parameters when high precision across multiple planes is not required (Stenum et al., 2021). Moreover, AI-based 2D-to-3D PE techniques are a fast-developing method to reduce these limitations, further expanding the potential applications of 2D systems (Fortini et al., 2023). We suggest that future studies aim to evaluate protocols and integrate vision sensors with PE framework software to bridge the gap in the development and validation of the 2D-to-3D PE device in real-world sports, thereby accessing biomedical data such as joint angles and providing real-time feedback.
